# Impact of the APE1 Redox Function Inhibitor E3330 in Non-Small Cell Lung Cancer Cells Exposed to Cisplatin: Increased Cytotoxicity and Impairment of Cell Migration and Invasion

**DOI:** 10.3390/antiox9060550

**Published:** 2020-06-24

**Authors:** Rita Manguinhas, Ana S. Fernandes, João G. Costa, Nuno Saraiva, Sérgio P. Camões, Nuno Gil, Rafael Rosell, Matilde Castro, Joana P. Miranda, Nuno G. Oliveira

**Affiliations:** 1 Research Institute for Medicines (iMed.ULisboa), Faculty of Pharmacy, Universidade de Lisboa, Av. Professor Gama Pinto, 1649-003 Lisboa, Portugal; rmanguinhas@campus.ul.pt (R.M.); sergiocamoes@campus.ul.pt (S.P.C.); mcastro@ff.ulisboa.pt (M.C.); jmiranda@ff.ulisboa.pt (J.P.M.); 2 Research Center for Biosciences & Health Technologies (CBIOS), Universidade Lusófona de Humanidades e Tecnologias, Campo Grande 376, 1749-024 Lisboa, Portugal; ana.fernandes@ulusofona.pt (A.S.F.); jgcosta@ulusofona.pt (J.G.C.); nuno.saraiva@ulusofona.pt (N.S.); 3 Lung Cancer Unit, Champalimaud Centre for the Unknown, Av. Brasília, 1400-038 Lisboa, Portugal; nuno.gil@fundacaochampalimaud.pt; 4 Laboratory of Cellular and Molecular Biology, Institute for Health Science Research Germans Trias i Pujol (IGTP), Campus Can Ruti, Ctra de Can Ruti, Camí de les Escoles, s/n, 08916 Badalona, Barcelona, Spain; rrosell@iconcologia.net; 5 Internal Medicine Department, Universitat Autónoma de Barcelona, Campus de la UAB, Plaça Cívica, 08193 Bellaterra, Barcelona, Spain

**Keywords:** non-small cell lung cancer, cisplatin, apurinic/apyrimidinic endonuclease 1, E3330, cytotoxicity, apoptosis, migration, invasion

## Abstract

Elevated expression levels of the apurinic/apyrimidinic endonuclease 1 (APE1) have been correlated with the more aggressive phenotypes and poor prognosis of non-small cell lung cancer (NSCLC). This study aimed to assess the impact of the inhibition of the redox function of APE1 with E3330 either alone or in combination with cisplatin in NSCLC cells. For this purpose, complementary endpoints focusing on cell viability, apoptosis, cell cycle distribution, and migration/invasion were studied. Cisplatin decreased the viability of H1975 cells in a time- and concentration-dependent manner, with IC_50_ values of 9.6 µM for crystal violet assay and 15.9 µM for 3-(4,5-Dimethylthiazol-2-yl)-5-(3-carboxymethoxyphenyl)-2-(4-sulfophenyl)-2H-tetrazolium (MTS) assay. E3330 was clearly cytotoxic for concentrations above 30 µM. The co-incubation of E3330 and cisplatin significantly decreased cell viability compared to cisplatin alone. Regarding cell cycle distribution, cisplatin led to an increase in sub-G1, whereas the co-treatment with E3330 did not change this profile, which was then confirmed in terms of % apoptotic cells. In addition, the combination of E3330 and cisplatin at low concentrations decreased collective and chemotactic migration, and also chemoinvasion, by reducing these capabilities up to 20%. Overall, these results point to E3330 as a promising compound to boost cisplatin therapy that warrants further investigation in NSCLC.

## 1. Introduction

**Figure 1:**
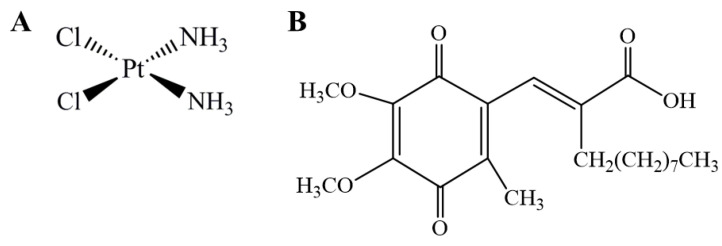
Chemical structure of cisplatin (**A**) and (2E)-2-[(4,5-dimethoxy-2-methyl-3,6-dioxo-1,4-cyclohexadien-1-yl)methylene] undecanoic acid (E3330) (**B**).

Worldwide, lung cancer (LC) is the first cause of cancer-related deaths and the most diagnosed type of cancer for men and women combined. In the US, LC is by far the main cause of death by cancer [1,2]. Non-small cell lung cancer (NSCLC) accounts for the majority of all lung cancer cases and has a low survival rate due to metastasis progression [3]. The most common chemotherapy regimens used in this type of cancer comprise the platinum-based drug cisplatin (Figure 1A), which exerts its effect by cross-linking DNA, inhibiting its replication and transcription, resulting in cell death [4,5]. Even though cisplatin is associated with slightly better survival rates, it is still associated with inherited and acquired resistance to therapy, making it inactive against some tumor types [6]. Indeed, cisplatin resistance is one of the major limitations to its clinical use [4]. Nevertheless, the mechanisms responsible for the resistance of tumor cells are not yet completely understood. For this reason, a number of possible mechanisms for cisplatin resistance were proposed, including reduced intracellular accumulation of cisplatin; enhanced drug inactivation by metallothionein and glutathione; altered expression of oncogenes and regulatory proteins; and increased repair activity of DNA damage [5]. The mechanistic findings on nicotine-induced cisplatin chemoresistance [7,8] should also be mentioned in the context of lung cancer in tobacco smokers. Moreover, both nicotine [9] and cigarette smoking [10] have been associated with oxidative stress.

The human apurinic/apyrimidinic endonuclease 1 (APE1) is an essential enzyme with two key functions, i.e., repairing DNA damage through the base excision repair (BER) pathway and also a redox signaling protein, modulating the activation of several transcription factors related to cell survival, proliferation, migration/invasion, inflammation, angiogenesis and metastases formation [11,12,13]. Several transcription factors have been related to APE1′s redox activity, particularly the nuclear factor-κB (NF-κB), activator protein 1 (AP-1), early growth response protein-1 (Egr-1), hypoxia-inducible factor 1α (HIF-1α), p53, signal transducer and activator of transcription 3 (STAT3), and Nuclear factor (erythroid-derived 2)-like 2 (Nrf-2) [12,14,15,16,17,18,19,20]. In addition, upon oxidative stress, APE1 is known to control the intracellular redox state via the inhibition of reactive oxygen species (ROS) production or by binding with transcription factors (such as p53, HIF-1α, and Nrf-2), promoting an antioxidant response [21,22,23]. Furthermore, multiple studies have demonstrated that APE1 is overexpressed in numerous types of cancer, such as NSCLC. Increased expression is also associated with more aggressive tumor phenotypes and poor prognosis [13,18,24]. For these reasons, APE1 has gained increasing attention as an emerging druggable target in cancer therapy. A study by Wang et al. [24] suggested that APE1 could be a promising target for the combination of cisplatin-based chemotherapy in NCSLC patients, since its total inhibition using siRNA enhanced sensitivity to cisplatin activity in A549 cells by enabling a synergistic relationship. Other complementary studies also demonstrated that the increased chemoresistance to cisplatin treatment could be related to APE1 overexpression up-regulating transcription factors related to cell survival, such as NF-κB and AP-1, and adaptive response to apoptotic stimulation [25,26,27]. All this evidence reinforces that APE1 plays a vital role as an upstream effector in cancer progression and reducing its expression levels could help with cisplatin therapy efficiency.

The quinone derivative (2E)-2-[(4,5-dimethoxy-2-methyl-3,6-dioxo-1,4-cyclohexadien-1-yl) methylene] undecanoic acid (E3330 or APX3330, Figure 1B) has been described as a direct and highly specific inhibitor of APE1 redox function, being the first to be identified [28]. For this reason, it has been tested to reduce growth-promoting, inflammatory and anti-apoptotic activities in cells, as well as tumor invasion and metastatic disease in different types of cancer [12,13,28,29]. Its therapeutic potential has been addressed in several cancer-cell-based studies, with interesting results including those from a previous study from our group with human breast cancer cells [13]. A study with prostate cancer also demonstrated important results by reducing cancer cell proliferation and inducing cell cycle arrest upon selective inhibition of the reduction-oxidation function with E3330 [30]. In pancreatic cancer, E3330 was able to impair tumor growth and blocked the activity of NF-κB, AP-1, and HIF-1α [31]. Furthermore, E3330 therapeutic efficacy has been shown to reduce the activity of the transcriptional activators, described previously, regulated by the APE1 redox activity [14,15,16,17]. As a result, E3330 has been evaluated in phase I clinical trials in patients with advanced solid tumors [29].

In this context, the present work aimed to assess the impact of APE1’s redox function inhibition by E3330 in NSCLC cells in vitro and if this active compound could improve the efficacy of cisplatin administration by enabling a synergistic effect. This evaluation was performed for the first time in the context of NSCLC by integrating multiple endpoints related to cell viability, cell cycle progression, apoptosis, and cell migration and invasion.

## 2. Materials and Methods

### 2.1. Chemicals

RPMI-1640 with l-glutamine was purchased from Biowest (Nuaillé, France). Cisplatin, E3330, penicillin-streptomycin solution (10,000 units/mL of penicillin; 10 mg/mL of streptomycin), crystal violet (CV), sodium bicarbonate, extracellular matrix (ECM) gel and dimethylsulfoxide (DMSO) were purchased from Sigma-Aldrich (Madrid, Spain). Sodium pyruvate was purchased from Lonza (Basel, Switzerland) and trypsin (0.25%), and fetal bovine serum (FBS) from Gibco (Eugene, OR, USA). Ethanol absolute, propidium iodide (PI), ethylenediaminetetraacetic acid (EDTA), and acetic acid were obtained from Merck (Darmstadt, Germany). HEPES and d-Glucose were purchased from AppliChem (Darmstadt, Germany). CellTiter 96^®^ Aqueous MTS (3-(4,5-Dimethylthiazol-2-yl)-5-(3-carboxymethoxyphenyl)-2-(4-sulfophenyl)-2H-tetrazolium) was acquired from Promega (Madison, WI, USA) and the Alexa Fluor 488^®^ Annexin V/PI Kit was acquired from Molecular Probes (Eugene, OR, USA).

A 25 mM stock solution of E3330 was prepared in DMSO, aliquoted, and stored at −20 °C. Working solutions were freshly diluted in complete cell culture medium so that, in each final solution, the DMSO concentration was kept at 0.2% (*v*/*v*). Cisplatin was dissolved in saline (0.9% NaCl) at a concentration of 2 mM, aliquoted, and stored at −20 °C. In all cell-based assays, vehicle-treated controls were also included, in which H1975 cells were exposed to the respective solvents, i.e., DMSO (final concentration of 0.2% (*v*/*v*)) or saline.

### 2.2. Cell Culture

The human NSCLC cell line H1975 was purchased from the American Type Culture Collection (ATCC, Manassas, VA, USA). This cell line was established in July 1988, originated from lung adenocarcinoma (NSCLC) and the tissue donor was a non-smoker female. H1975 cells were cultured in monolayer in RPMI-1640 medium with l-glutamine supplemented with 10 mM HEPES, 2.5 g/L d-glucose, 1 mM Sodium pyruvate, 1.5 g/L Sodium bicarbonate, 10% FBS and 1% Pen/Strep (complete cell culture medium) and were maintained at 37 °C, under a humidified atmosphere containing 5% CO_2_ in air.

### 2.3. Crystal Violet (CV) Staining Assay

The cytotoxicity of cisplatin/E3330 alone or in combination, in H1975 cells, was evaluated according to a previously described CV staining protocol [13]. Cells were seeded for 24 h at a density of approximately 3 × 10^3^ cells/well in 200 μL of complete culture medium in 96-well plates. After the 24 h period, culture medium was changed and the cells were incubated with a range of cisplatin (1–50 µM) or E3330 (5–50 µM) concentrations, for another 72 h (and also 48 h for cisplatin), in order to assess concentration-response profiles. For combinatory assays, cells were pre-incubated with E3330 (30 µM) for 3 h and then simultaneously exposed to E3330 and different concentrations of cisplatin (5–20 µM) for 72 h. After the incubation periods, H1975 cells were washed with phosphate-buffered saline (PBS) to remove non-adherent cells (non-viable cells). The adherent cells were then fixed with ice-cold 96% ethanol for 10 min and stained with 0.1% crystal violet in 10% ethanol for 5 min. After rinsing with tap water, the stained cells were dissolved in 200 µL of 96% ethanol with 1% acetic acid. Absorbance was measured at 595 nm (OD595) using a SPECTROstar OMEGA microplate reader (BMG Labtech, Offenburg, Germany). Absorbance values presented by vehicle-treated control cells (untreated cells) corresponded to 100% of cell viability. Three to five independent experiments were carried out and six replicates were used for each condition in each independent experiment. The IC_50_ was calculated based on the concentration–response curve using GraphPad Prism^®^ 7.0 (GraphPad Software, Inc., La Jolla, CA, USA).

### 2.4. MTS Reduction Assay

The MTS reduction assay was carried out as a confirmatory assay of cell viability by applying the same experimental conditions as in the CV assay and following an already described protocol [13]. Briefly, after treatment with the compounds and removal of the incubation medium, cells were washed with PBS, followed by the addition of 100 μL of fresh complete growth medium plus 20 μL of MTS substrate prepared from the CellTiter 96^®^ Aqueous MTS, according to the manufacturer’s instructions. Cells were incubated for 2 h with the MTS reagent and the results were measured in terms of absorbance at 490 nm and 690 nm (reference wavelength) using a SPECTROstar OMEGA microplate reader (BMG Labtech, Offenburg, Germany). Absorbance values presented by vehicle-treated control cells corresponded to 100% of cell viability. Three to four independent experiments were carried out and three replicates were used for each condition in each independent experiment. The IC_50_ was also calculated based on the concentration–response curve using GraphPad Prism^®^ 7.0 (GraphPad Software, Inc., La Jolla, CA, USA).

### 2.5. Cell DNA Content Analysis

Cell DNA content analysis by flow cytometry was performed in order to evaluate the effect of the combination of both compounds in cell cycle distribution. This procedure was carried out according to previously described protocols [32,33]. Briefly, 6 × 10^4^ cells/well were seeded in 6-well plates and cultured for 21 h. Afterwards, cells were incubated with E3330 (30 μM) for 3 h. The culture medium was then changed and both cisplatin (20 μM) and E3330 (30 μM) were added to the cells and incubated for an additional 72-h period. Cells were harvested using 5 mM EDTA in PBS at 37 °C, washed with cold PBS and fixed with chilled 80% ethanol. Cells were stained with PI (10 μg/mL) and simultaneously treated with RNase A (20 μg/mL) for 15−20 min. PI staining was analyzed using a FACSCalibur flow cytometer (Becton Dickinson, San Jose, CA, USA). Data acquisition and analysis were performed using CellQuest^®^ software (Becton Dickinson, San Jose, CA, USA ) and FlowJo^®^ (Tree Star Inc., San Carlos, CA, USA), respectively. Three independent experiments were performed.

### 2.6. Apoptosis Assay

The percentage of apoptotic cells was measured using the flow cytometry dead cell apoptosis kit with Alexa^®^ Fluor 488 Annexin V and PI according to the manufacturers’ instructions (Molecular Probes, Eugene, OR, USA). Roughly, 9 × 10^4^ cells were seeded in 6-well plates and grown for 21 h at 37 °C in complete growth medium. After the 21 h period, E3330 (30 μM) was added to the cells for 3 h (pre-incubation). The culture medium was then renewed and both cisplatin (20 μM) and E3330 (30 μM) were added to the cells and incubated for further 72 h. Afterwards, the cells were detached with 5 mM EDTA in PBS at 37 °C and washed with cold PBS. Cells were then stained with PI and Alexa Fluor^®^ 488 annexin V according to the manufacturer’s instructions and analysed using a FACSCalibur flow cytometer (Becton Dickinson, San Jose, CA, USA). Three independent experiments were performed. Data acquisition and analysis were performed using CellQuest^®^ software (Becton Dickinson, San Jose, CA, USA) and FlowJo^®^ (Tree Star Inc., San Carlos, CA, USA), respectively.

### 2.7. Selection of Cisplatin and E3330 Concentrations for the Migration/Invasion Assays

To select non-toxic concentrations of both compounds for the migration and invasion assays, CV and MTS assays were performed as described previously, but using medium with a low serum content instead. This step is of utmost importance to avoid misleading conclusions in migration assays due to cytotoxic effects. Taking this into account, 8 × 10^3^ cells/well were seeded in 96-well plates in complete culture medium containing 10% FBS for 24 h. After the 24 h period, the complete cell culture medium was replaced by culture medium containing 2% FBS and cells were then incubated with a range of low concentrations of cisplatin (0.1−5 μM) or E3330 (10−30 μM) for another 24 h. The CV and MTS assays were subsequently performed and non-toxic concentrations of both E3330 and cisplatin were selected for the following assays. Three to seven independent experiments were performed, each one comprising three (MTS) or six (CV) replicates.

### 2.8. In Vitro Wound-Healing Assay

For the evaluation of collective cell migration, an in vitro wound-healing assay was performed according to a previously described method [34,35]. H1975 cells were seeded in 24-well plates at a density of approximately 5.5 × 10^4^ cells/well and incubated for 21 h in complete cell culture medium. E3330 was then added to the well at a concentration of 10 μM and incubated for 3 h. Afterwards, the cell culture medium was removed, and a scratch was performed using a 200 μL sterile pipette tip on the cell monolayer. Cells were then washed twice with warm PBS, in order to remove cellular debris, and were left to migrate in cell culture medium containing 2% FBS in the presence of cisplatin (1 μM) and E3330 (10 μM) for further 20 h. Wound closure was evaluated using a Motic AE2000 Inverted Phase Contrast Microscope (Motic, Barcelona, Spain) and pictures of the same areas were captured using a magnification of 40× with a camera Moticam 2500 (Motic, Barcelona, Spain). The scratch width was measured with Motic Images plus v2.0 software (Motic, Barcelona, Spain) at 0, 8, and 20 h after the scratch was performed. The percentage of cell migration was measured in relation to the initial distance between the wound edges. At each time-point, two pictures of the scratch were taken for each condition. Three independent experiments were performed.

### 2.9. Chemotaxis and Chemoinvasion Assays

As a single-cell migration evaluation, a chemotactic migration assay was performed by adopting a protocol already described by Flórido et al. [36] and Fernandes et al. [34]. Briefly, 3 × 10^4^ cells/well were seeded in cell culture medium containing 2% FBS on the top of a transwell insert with transparent polyethylene terephthalate (PET) membranes containing 8 μm pores (BD Falcon, Bedford, MA, USA) inside 24-well plates. Complete cell culture medium was added to the lower chamber, containing 10% FBS as the chemoattractant. Right after seeding, E3330 (10 μM) alone was added to both chambers and incubated for 3 h. After this 3 h period, cisplatin (1 μM) was also added to both chambers and cells were allowed to migrate through the membrane for another 16 h. Subsequently, non-migrating cells were carefully removed from the upper chamber with a cotton swab and migrating cells (bottom of each membrane) were fixed with cold 96% ethanol for 10 min and then stained with 0.1% crystal violet in 10% ethanol for 15 min. The inserts were thoroughly rinsed using tap water and were allowed to dry for at least 24 h. Five randomly selected fields were photographed for each condition, using a Moticam 2500 (Motic, Barcelona, Spain) placed on a Motic AE2000 Inverted Phase Contrast Microscope (Motic, Barcelona, Spain) with an amplification of 100×. For each picture, migrated cells were manually counted using the software Motic Images plus v3.0 (Motic, Barcelona, Spain). The counted cells were expressed as percentages of vehicle-treated control cells and three independent experiments were performed. 

A procedure similar to the abovementioned chemotactic migration assay was carried out for the evaluation of chemoinvasion. The difference between the two setups was the addition of 75 μL of ECM gel (1:25 dilution in serum-free medium) in order to coat the porous membranes of the transwell inserts. The initial seeding density was also adapted to 1.5 × 10^4^ cells/well. The analysis of the results was performed similarly to the abovementioned chemotactic migration assay, and five independent experiments were performed.

## 3. Results

### 3.1. Cytotoxicity Profile of Cisplatin in H1975 Cells

**Figure 2:**
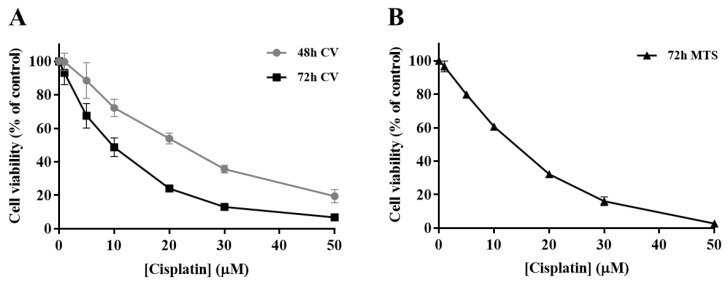
Cytotoxic effects of cisplatin (1–50 µM) in H1975 cells. The viability of cells treated with cisplatin for 48 h and 72 h was assessed by crystal violet (CV) staining assay (**A**) and for 72 h by MTS reduction assay (**B**). Values represent mean ± SD (*n* = 3–4) and are expressed as percentages of the vehicle-treated control cells.

In order to determine the impact of cisplatin treatment in H1975 cells viability, a concentration-response profile was established, resorting to CV staining and MTS reduction assays. In the CV staining assay, after 48 and 72 h of cisplatin exposure (Figure 2A), the viability of H1975 cells decreased in a time- and concentration-dependent manner (1–50 µM). Additionally, The MTS reduction assay (Figure 2B) was performed as a mechanistically complementary method and the concentration–response curves showed similar cytotoxicity profiles. The IC_50_ values for the CV assay were 27.5 and 9.6 µM for 48 and 72 h, respectively. In addition, the IC_50_ value calculated for the MTS assay at 72 h was 15.9 µM, in the same range, although slightly higher. Cisplatin was demonstrated to be toxic at low concentrations for a 72 h incubation period, starting to compromise cell viability at 1 µM and dramatically decreasing it at 50 µM, for both MTS and CV assays. Considering these results, the cisplatin concentrations of 5, 10, and 20 µM were selected for the subsequent combinatory assays, since they represent different levels of cytotoxicity, comprising the range of IC_50_ values calculated as well as concentrations slightly above and below this parameter.

### 3.2. Impact of E3330 in the Viability of H1975 Cells

**Figure 3:**
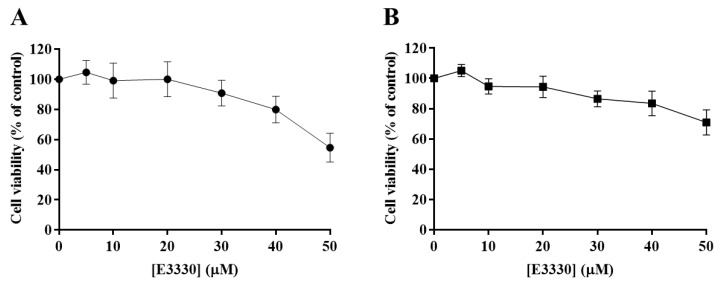
Evaluation of E3330 (5–50 µM) cytotoxicity in H1975 cells. The cell viability of E3330-exposed cells (72 h) was evaluated by CV staining (**A**) and MTS reduction (**B**) assays. Values represent mean ± SD (*n* = 3) and are expressed as percentages of the vehicle-treated control cells.

The effect of E3330 was evaluated by exposing H1975 cells during 72 h to a range of concentrations from 5 to 50 µM. Both CV and MTS assays revealed that E3330 was not considerably toxic at low concentrations (Figure 3A,B, respectively). Both assays demonstrated a similar concentration–response curve for E3330. Nevertheless, E3330 at 50 µM showed decreased cell viability in about 45% with the CV assay whereas, with the MTS assay, the decrease was lower, approximately 30%. A similar trend in the differences between these two methods was also observed in the previous cisplatin assays, reflecting the inherent sensitivities of these two mechanistically distinct endpoints. Since the range of E3330 concentrations applied for these experimental conditions did not lead to a 50% loss in cell viability, it was not possible to calculate the IC_50_ values for H1975 cells. The concentration of 30 µM was chosen for the combinatory assays since it was the higher concentration of E3330 tested that displayed a relatively low impact on cell viability.

### 3.3. The Combination of E3330 and Cisplatin Displays a Synergistic Effect in Cell Viability

**Figure 4:**
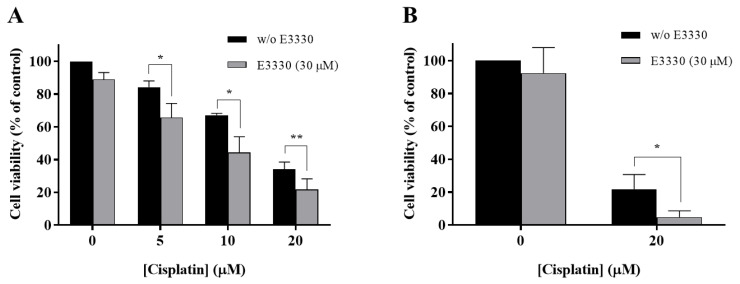
Impact of E3330 on the viability of H1975 cells treated with cisplatin. Cells were pre-incubated with E3330 (30 µM) for 3 h and then simultaneously exposed to E3330 and cisplatin (5–20 µM) for 72 h. The effects in terms of cell viability were evaluated using the CV staining assay (**A**) and MTS reduction assay (**B**). Values represent mean ± SD (*n* = 3–5) and are expressed as percentages relative to vehicle-treated control cells. * *p* < 0.05 and ** *p* < 0.01 relative to respective cisplatin-treated cells (Student’s *t*-test).

With the purpose of evaluating if E3330 enhanced cisplatin treatment in NSCLC, H1975 cells were co-incubated with these two compounds and the effects were evaluated using the CV staining assay and validated with the MTS reduction assay. In the CV assay, E3330 (30 µM) demonstrated a slight decrease in cell viability of around 11% (*p* < 0.01) when compared to the vehicle-treated control cells (Figure 4A). In the MTS assay, this decrease was lower and not statistically significant (Figure 4B). All the concentrations of cisplatin (5, 10, and 20 µM) tested in the CV assay revealed an impairment in cell viability that was clearly intensified when the APE1 redox inhibitor E3330 was co-incubated. This significant combined effect was also confirmed in the MTS assay. In this case, the cells were treated with 20 µM of cisplatin and 30 µM of E3330. In absolute percentage values, the decreases in cell viability observed for 5, 10 and 20 µM of cisplatin, in the presence of E3330, were 18.5% (*p* < 0.05), 22.8% (*p* < 0.05) and 12.4% (*p* < 0.01), respectively, for the CV assay, and 17.1% (*p* < 0.05) for the MTS assay. Considering the relative decreases in cell viability observed, the concentration of E3330 at 30 µM reduced in 36% and 78% the cell viability of 20 µM cisplatin-treated cells for the CV and MTS assays, respectively. As such, this combination was selected for further cell cycle distribution studies. Altogether, these results suggest that for all the concentrations and endpoints tested, a synergistic effect was present.

### 3.4. Effect of the Combination of E3330 and Cisplatin in Cell Cycle Distribution and Cell Death

**Figure 5:**
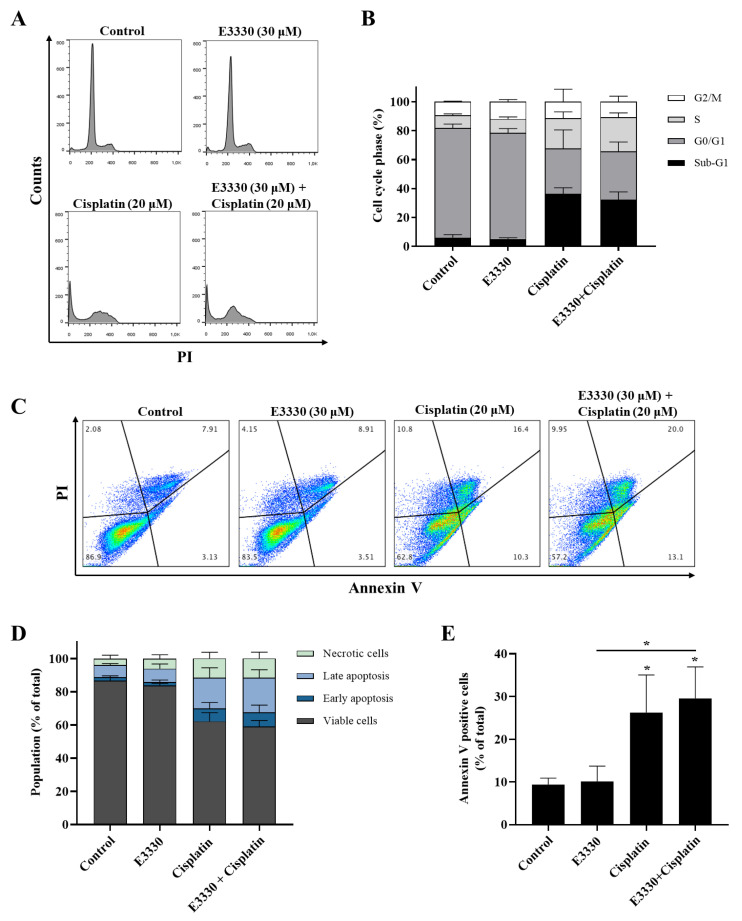
Cell cycle progression and apoptosis of H1975 cells treated with E3330 and/or cisplatin. Cells were pre-incubated with E3330 (30 µM) for 3 h and then cisplatin (20 µM) was added for co-incubation for further 72 h. After this exposure period, cell DNA content analysis with PI staining was performed by flow cytometry. (**A**) Representative flow cytometry histograms. (**B**) Sub-G1, G0/G1, S, and G2/M populations’ summary results. The percentage of apoptotic cells was determined by PI and Annexin V staining after the same incubation profile as in the cell DNA content analysis. (**C**) Representative flow cytometry dot-plots. (**D**) Percentage of viable cells, cells undergoing early and late apoptosis, and necrotic cells summary results. (**E**) Summary results demonstrate the percentage of apoptotic cells (Annexin V positive cells). Values represent mean ± SD (*n* = 3), * *p* < 0.05 (one-way ANOVA with Tukey’s test).

Since cytotoxicity is frequently accompanied by cell cycle arrest and/or cell death, the impact of E3330 and cisplatin combination in H1975 cells was evaluated by cell DNA content using PI staining for flow cytometry (Figure 5A,B). As expected, the exposure to cisplatin (20 μM, 72 h) alone substantially increased (around five-fold) the sub-G1 population and lead to a decrease in the G0/G1 population when compared to vehicle-treated control cells. E3330 (30 μM, 72 h) did not significantly modify the cell cycle distribution of vehicle-treated or cisplatin-treated cells. In fact, the cell cycle distribution of cisplatin alone or cisplatin with E3330 remained similar. G2/M population maintained unaltered for all the conditions tested.

The induction of apoptosis was analyzed by flow cytometry after staining with Annexin V-FITC and PI (Figure 5C–E). Representative graphs obtained by flow cytometry are displayed in Figure 5C. Incubation with cisplatin (20 μM, 72 h) alone led to a ~3-fold increase in the % of apoptotic cells when compared to vehicle-treated cells (Figure 5E; ~26% vs 9%, respectively), which is in line with the observed increase in the sub-G1 population. Moreover, although the exposure to E3330 did not significantly alter the % of apoptotic cells in cisplatin-treated cultures, a small trend towards a synergistic effect was observed (Figure 5E).

### 3.5. E3330 in Combination with Cisplatin Reduces Both Collective and Chemotactic Cell Migration

**Figure 6:**
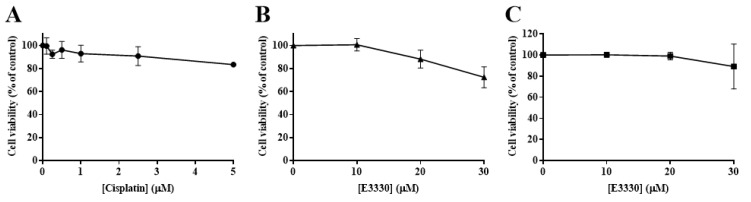
Viability of H1975 cells exposed to low concentrations of cisplatin or E3330 in culture medium with 2% FBS. (**A**) Effect of cisplatin (0.1–5 μM; 24 h) on cell viability, in the presence of 2% FBS, evaluated by the CV assay. Effect of E3330 (10–30 μM; 24 h) on cell viability in the presence of 2% FBS evaluated by both CV (**B**) and MTS assays (**C**). Values for cell viability represent mean ± SD (*n* = 4–7) and are expressed as percentages relative to vehicle-treated control cells.

Considering an impairment in cell viability would interfere with possible results in cell migration and invasion processes, it was necessary to ascertain that non-toxic concentrations of E3330 and cisplatin were used. As such, the H1975 cells were exposed to a range of low concentrations of either cisplatin (0.1–5 μM) or E3330 (10–30 μM) for 24 h in order to select the conditions of migration/invasion assays. Accordingly, in these experiments, a complete culture medium with 2% FBS was used. The effect of cisplatin was assessed using the CV assay (Figure 6A). Cell viability was not markedly affected up to 2.5 µM (~10% reduction). At a concentration of 5 μM, this decrease reached around 17%. As for E3330, cell viability started to be affected at the concentration of 20 μM with a reduction of 12% in the CV assay (Figure 6B), although in the MTS assay this concentration level was not cytotoxic (Figure 6C). Overall, considering both assays and compounds, we decided to select the representative non-cytotoxic concentrations of 1 μM for cisplatin and 10 μM for E3330 for the migration and invasion assays.

**Figure 7:**
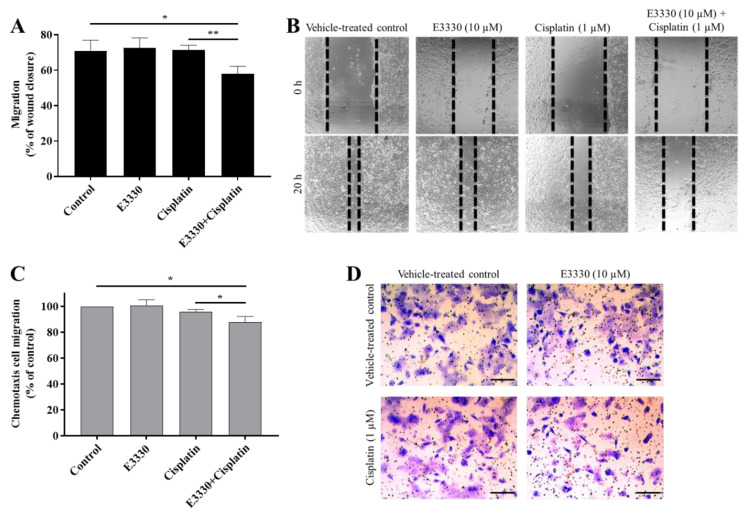
E3330 effect on collective and chemotactic migration of H1975 cells exposed to cisplatin. Collective cell migration was evaluated by the wound-healing assay (**A**) and chemotaxis was measured using a transwell assay (**C**). Representative microscopy images of the wound-healing assay (40×, **B**) and the chemotaxis assay (migrating cells stained with crystal violet—100×, **D**). Scale bars = 200 μm. Values for the wound-healing assay represent mean ± SD (*n* = 3) and are expressed as percentage of wound closure, calculated relative to the initial width; * *p* < 0.05 and ** *p* < 0.01 (Student’s *t*-test). Values for the chemotaxis assay represent mean ± SD (*n* = 3) and are expressed as percentages relative to vehicle-treated control cells; * *p* < 0.05 (Student’s *t*-test).

Metastases development comprises multiple biological mechanisms, including an increase in cell motility. For this reason, after the selection of non-cytotoxic concentrations of both E3330 and cisplatin, the migration capacity of H1975 cells was evaluated by resorting to two mechanistically different methods. Firstly, collective cell migration was assessed with the wound-healing assay as an evaluation of the cells’ movement across a horizontal surface with the conservation of functional cell–cell junctions (Figure 7A). Both E3330 and cisplatin alone did not demonstrate an effect on wound closure. Importantly, their combination significantly reduced this closure in about 20% (*p* < 0.05) when compared to vehicle-treated control cells. This decrease was also statistically significant when comparing the combination of both compounds with cisplatin-treated cells (*p* < 0.01). Microphotographs were also taken at a timepoint of 8 h of co-incubation, already revealing a slight impairment for the combinatory condition (data not shown). 

For the determination of chemotactic individual cell migration, the transwell assay was performed (Figure 7C) as a measurement of the ability of single cells to directionally respond to a chemoattractant gradient. In this assay, E3330 and cisplatin alone also did not influence chemotactic migration, but again their combination reduced this type of migration in approximately 12% (*p* < 0.05) when compared to vehicle-treated control cells. The decrease observed was also statistically significant when compared to cisplatin-treated H1975 cells (*p* < 0.05). Representative images of the wound-healing assay and the chemotaxis migration assay are presented in Figure 7B,D, respectively.

### 3.6. The Combination of E3330 and Cisplatin Decreases Invasion of H1975 Cells

**Figure 8:**
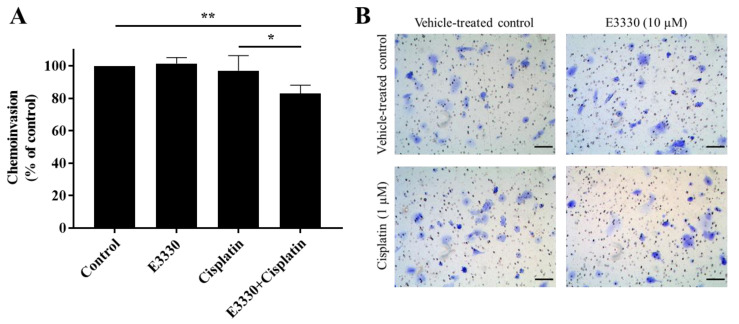
Effect of E3330 on the invasiveness of cisplatin-treated H1975 cells. (**A**) Transwell chemoinvasion was assessed after a pre-incubation period of 3 h with E3330 and a subsequent period of 16 h with both compounds. Values represent mean ± SD (*n* = 5) and are expressed as percentages relative to vehicle-treated control cells; * *p* < 0.05 and ** *p* < 0.01 (Student’s *t*-test). (**B**) Representative microscopy images of invading cells stained with crystal violet (100×). Scale bars = 100 μm.

For the progression of cancer and metastases formation, cancerous cells located in the primary tumor need to be able to invade through the extracellular matrix and consequently migrate throughout blood circulation and/or lymphatic vessels and attach to a distant site in response to a stimulus. Considering these processes, the effect of E3330 on the invasion of cisplatin-treated H1975 cells was evaluated by the transwell chemoinvasion assay (Figure 8A). Since the proteolytic degradation of basement membranes is essential for invasion processes and subsequent metastasis formation, this assay was performed under the same conditions as the chemotaxis migration assay but with the incorporation of an ECM gel. Similar to the results from the migration assays, both E3330 and cisplatin alone did not induce a significant effect in terms of cell invasiveness. However, when both compounds were combined, there was a statistically significant decrease of approximately 17% (*p* < 0.01) in chemoinvasion when compared to vehicle-treated control cells. This decrease was also statistically significant when comparing the combination of compounds with cisplatin-treated H1975 cells (*p* < 0.05). Representative images of the chemoinvasion assay are presented in Figure 8B.

## 4. Discussion

As aforementioned, NSCLC is the most frequent lung cancer sub-type, presenting low survival rates due to metastasis progression, which is often resistant to platinum-based chemotherapy. The usefulness of genetics to predict the response of cisplatin was reviewed by Karachaliou et al. [37]. Rosell et al. [38] also recently highlighted the novel molecular targets for the treatment of NSCLC, identifying different prognostic markers. In fact, there are important molecular markers that should be considered in NSCLC. For instance, HIF-1α was reported as a prognostic factor for lung cancer patients [39]. In the scope of the present study, and as described in the Introduction Section, the elevated expression levels of APE1 have also been correlated with more aggressive phenotypes and poor prognosis of NSCLC patients. 

APE1, besides being a key DNA repair enzyme, also works as a redox signaling protein, modulating the activation of several transcription factors related to cancer progression and metastasis formation. For this reason, the aim of the present work was to address in vitro the impact of a novel therapeutic strategy based on targeting APE1 redox function in NSCLC in order to increase the efficacy of platinum-based chemotherapy and reduce its possible resistance. This innovative approach constitutes the first report on the effect of E3330 alone or in combination with cisplatin in NSCLC cells using complementary endpoints. The representative cell model chosen for this purpose is the H1975 human lung adenocarcinoma cell line that was established from a non-smoker patient and possesses a mutation in the gene that confers resistance to EGFR inhibitors [40]. It is considered as a highly invasive cell line used as an adequate tool in preclinical studies towards the discovery of novel drugs for NSCLC and also in xenograft models [41]. 

According to two complementary cell viability assays, cisplatin displayed a concentration- and time-dependent cytotoxic effect with IC_50_ values ranging from approximately 10 to 16 µM for 72 h, being more than two-fold higher for a shorter incubation period of 48 h. Considering the results available in the literature, the effect of cisplatin in H1975 cells viability herein obtained is comparable to those described by other authors using different methodologies such as sulforhodamine B assay (IC_50_ = 8.31 µM [27]) and MTT (3-(4,5-dimethylthiazol-2-yl)-2,5-diphenyltetrazolium bromide) assay (IC_50_ = 6.71 µM [42] and IC_50_ = 11 µM [43]) for the same 72 h incubation period. As for 48 h of cisplatin incubation in the same cell line, our results differ from the study of Zhao et al. [44] that found an IC_50_ value of 3 µM using the MTT assay. In contrast, Sun et al. [45] with the Cell Counting kit-8 assay, obtained an IC_50_ value of around 30 µM for H1975 cells, which was comparable to our results. It is known that cell viability for similar cisplatin concentrations varies among different cell lines, even for cells originating from the same NSCLC cancer subtype, due to various reasons including acquired cisplatin resistance. For example, A549 cells appear to be more sensitive to cisplatin incubation (72 h) than the H1975 cells as shown by Wang et al. (IC_50_ = 1.54 µM [24]), Deben et al. (IC_50_ = 4.12 µM [27]) and Wang et al. (IC_50_ = 5.7 µM [43]). In contrast, H1993 is a NSCLC cell line more resistant to cisplatin, with an IC_50_ value of 19.58 µM [46]. In this sense, the choice for H1975 cells was considered here in view of its average sensitivity to cisplatin effects. 

In the present work, E3330 did not induce significant cytotoxicity at low concentrations in H1975 cells. However, this compound has demonstrated a significant impact on different cancer cell lines. At the same incubation period, and with an MTT assay, E3330 was demonstrated to be more cytotoxic to ovarian cancer cell lines with IC_50_ values of 33 µM and 37 µM in Hey-C2 and SKOC-3X cell lines, respectively [47]. In the case of prostatic cancer, McIlwain et al. [30] presented the following IC_50_ values for a five day incubation period with E3330: PC-3, 54.7 µM; C4-2, 89.5 µM, and LNCaP, 71.9 µM. As for pancreatic cancer, E3330 cytotoxicity was demonstrated to fluctuate between different cell lines by presenting an IC_50_ of 50 µM for PANC1 cells [48] and causing only 20% loss of cell viability at the same concentration in Pa03C cells [19]. However, this compound has only been tested in a NSCLC cell line (A549) with a concentration of 25 µM for 72 h, resulting in less than 5% loss in cell viability [49], which was similar to the results obtained in the present work. Based on these results, it can be concluded that E3330 is not considerably cytotoxic to H1975 cells at concentrations up to 30-40 µM, displaying only a clear cytotoxic effect at 50 µM. Interestingly, the blood levels found in clinical trials varied between 50 and 150 µM [30], higher than the range of E3330 concentrations that were effective in the combinatory experiments with cisplatin described in the present study.

Importantly, when both drugs were combined in the CV and MTS assays, a significant decrease in cell viability was revealed. Since cytotoxicity is frequently accompanied by cell cycle arrest and apoptosis induction, we aimed to explore this synergistic relationship between E3330 and cisplatin in terms of cell cycle distribution. However, the addition of E3330 did not alter the profile of H1975 cisplatin-treated cells. Moreover, E3330 per se did not have an impact on the cell cycle distribution. This could be a consequence of its low cytotoxic potential (Figure 3). Similar results were also observed in representative cell lines of pancreatic cancer (PANC1 [48]) and breast cancer (MDA-MB-231 [13]). The cytotoxicity of cisplatin is mediated through the induction of apoptosis and cell cycle arrest resulting from its interaction with DNA [5]. It has been demonstrated that cisplatin can affect the Gl-S checkpoint, when the cell is entering the S-phase, or the G2/M checkpoint, after DNA replication [50,51]. For example, in a study with A549 cells, it was demonstrated that an incubation with 11 μM of cisplatin for 24 h increased the G2/M phase population (cell cycle arrest) and a decrease in G0/G1 population [52]. This impact is also observed in our results. Considering the results achieved in the cytotoxicity assays, the evaluation of apoptosis was also performed. As expected, the incubation of cisplatin alone induced a high increase in the number of apoptotic cells. However, E3330 did not significantly increase the apoptosis induced in cisplatin-treated cells. Taking this into consideration, other cell mechanisms could be involved in promoting the loss of cell viability when E3330 and cisplatin are combined.

The ability of cells to migrate and invade surrounding tissues is essential for the development of metastases, and APE1′s redox function has been shown to modulate several transcription factors and signaling pathways related to these mechanisms [11,12]. Since E3330 inhibits this redox function, the combination of this compound with cisplatin was evaluated on these processes by assessing two mechanistically different migration endpoints and performing the reference chemoinvasion assay. E3330 and cisplatin alone did not interfere with both migration and invasion endpoints tested. However, when these compounds were combined, both collective and chemotactic cell migration, and also chemoinvasion, were reduced in levels up to 20%, this being also a relevant finding of the present study. It should be noted that higher concentrations of E3330 would likely lead to more pronounced results. However, the use of high concentrations of a given anti-migratory drug may enclose cytotoxicity, thus precluding the accurate assessment of migration. In addition, Nyland et al. demonstrated that in order to have redox inhibition by E3330, in ovarian Hey-C2 cells, a concentration of 10 μM was sufficient [53]. The use of E3330 potential to interfere with migration has been evaluated by other authors in different cancer models. For instance, E3330 impaired migration in pancreatic cancer cells [48] and retinal endothelial cells [54]. A previous study from our group using breast cancer cells (MDA-MB-231) also demonstrated a significant decrease in collective cell migration but not in chemotaxis [13]. Additionally, E3330 promoted a significant decrease in chemoinvasion when combined with docetaxel, a standard chemotherapeutic drug for breast cancer.

Overall, it is clear that the combination of E3330 and cisplatin is able to sensitize NSCLC cells, promoting a better response in terms of cytotoxicity and cell migration and invasion. A previous study by Li et al. showed that NF-κB expression is linked to chemoresistance in NSCLC cells (H460 cells), and consequent inhibition of this transcription factor enhances the sensitivity of these cells to cisplatin [55]. Furthermore, NF-κB, and also AP-1, have been associated with several downstream mechanisms related to cell migration and invasion such as matrix metalloproteinases (MMPs) and the hyaluronan cell-surface receptor CD44 activity. E3330 was able to suppress CD44 expression in pancreatic cancer cells, promoting a direct consequence for cell migration [48]. Besides, overexpression of this receptor has been correlated with occurrence and migration of NSCLC [56]. MMPs have also been established as key players in mechanisms of tumor invasion and metastasis formation by ECM degradation and are regulated by AP-1 and NF-κB, being MMP-9 strongly regulated by the latter [57,58]. In addition, this MMP has been found to be upregulated in NSCLC [59]. 

Altogether, the blocked transcription factors and subsequent downstream effectors may be responsible for the abovementioned effects in cytotoxicity and the reduction in migration and invasion upon treatment with both compounds. In this sense, the evaluation of the role of transcriptional factors constitutes a further step of this work in order to elucidate the mechanisms involved. This study should be performed in a holistic manner, focusing on a set of putative key targets (e.g., AP-1, Nrf2, NF-κB, HIF-1α). In accordance, it should be also pertinent to perform experiments to gain insights on other unknown transcriptional targets, resorting to RNA sequencing by Next Generation Sequencing (NGS) followed by protein expression confirmation. The inclusion of in vivo animal data is also anticipated after further elucidation of the putative mechanisms. There are several NSCLC rodent models available, including patient-derived xenografts, which can be adequate to study the combination of cisplatin (i.v.) with E3330 orally administrated. Finally, it should be emphasized that E3330 has been tested in cancer clinical trials for solid tumors (oral administration, twice a day). In view of this, the collection of in vitro and in vivo data will be determinant to support the potential clinical use of this drug in combination with standard platinum-based therapy in NSCLC.

## 5. Conclusions

The work developed herein enabled us to evaluate the impact of the APE1 redox inhibitor E3330 in H1975 cells treated with cisplatin by characterizing the cytotoxicity, cell cycle distribution, apoptosis, and migration and invasion processes. Overall, the results pointed to E3330 as a promising compound to boost cisplatin therapy that warrants further investigation in NSCLC. The results highlight that additional studies should be performed to elucidate the underlying mechanisms involved, such as verifying the expression of transcription factors under APE1 redox function which are also related to cell migration and invasion.
